# The *MTNR1B* Rs724030 variant is associated with islet function and women waist-to-hip ratio in healthy subjects

**DOI:** 10.3389/fendo.2024.1398687

**Published:** 2025-01-16

**Authors:** Sijie Zhang, Wenxuan Bian, Yan Wang, Min Shen, Yu Qian, Hao Dai, Shuai Zheng, Qi Fu, Kuanfeng Xu, Tao Yang, Hemin Jiang

**Affiliations:** Department of Endocrinology and Metabolism, The First Affiliated Hospital of Nanjing Medical University, Nanjing, China

**Keywords:** MTNR1B, variant, prediabetes, islet function, sex dimorphisms

## Abstract

**Objective:**

This study aims to investigate the associations between *MTNR1B* rs724030 A>G variant and prediabetes risk, along with their correlations with clinical features, including plasma glucose and serum insulin levels during oral glucose tolerance test (OGTT), islet function, insulin resistance, and plasma lipid levels. In particular, we investigated whether there are sex dimorphisms in the impact of this variant on islet function/insulin resistance.

**Methods:**

We included 3415 glucose-tolerant healthy and 1744 prediabetes individuals based on OGTT. Binary logistic regression was performed to evaluate the relationships between rs724030 in *MTNR1B* and prediabetes under the additive model. Additionally, multiple linear regression was utilized to investigate the associations between this variant and glycemic-related quantitative traits and lipid levels.

**Results:**

While no association was observed between the rs724030 variant in *MTNR1B* and prediabetes risk in the overall cohort (P > 0.05), we found the G allele of this variant was associated with higher fasting and 30-minute plasma glucose levels, decreased Insulinogenic Index (IGI), and oral disposition index (DIo) (P = 0.009, 0.001, 0.001, and 0.007, respectively) in the normal glucose tolerance (NGT) individuals with normal BMI levels. Furthermore, we also found significant associations between this variant and IGI, corrected insulin response (CIR), and DIo (All P < 0.001) in female individuals whose waist-to-hip ratio (WHR) is greater than 0.85, with considerable heterogeneity (P_het_ = 0.009, 0.030, and 0.049, respectively) to male participants in the NGT individuals, but not in the impaired fasting glucose (IFG)/impaired glucose tolerance (IGT) individuals. Additionally, no association was observed between this variant and insulin clearance (All P > 0.05).

**Conclusions:**

The *MTNR1B* rs724030 variant contributes to glycemic traits and islet function, and its effects have sex dimorphisms in the NGT individuals after stratifying by WHR. All these findings provide a basis for accurately assessing islet function in healthy populations and offer a new perspective on precision prevention.

## Introduction

The increasing prevalence of type 2 diabetes mellitus (T2DM) has become a primary global public health concern in recent years. In 2019, there were 463 million people with diabetes; by 2045, this number is expected to rise by 51%, reaching 700 million (1). Prediabetes is a condition in which plasma glucose levels are elevated but not high enough to be diagnosed as diabetes. According to the World Health Organization (WHO) criteria, IGT (2-h glucose 7.8-11.0 mmol/L) and IFG (fasting glucose 6.1-6.9 mmol/L) are used to define prediabetes (2). Previous studies found that the prevalence of prediabetes is gradually increasing and reached 38.1% in 2018 in China (3). Approximately 10% of individuals with prediabetes progress to diabetes annually in the U.S. Prediabetes is a significant risk factor for the development of diabetes, cardiovascular events, and increased mortality (4). Therefore, early detection and management through lifestyle changes, medication, and regular monitoring can prevent or delay diabetes onset and its complications.

Genome-wide association studies (GWAS) have been used to identify links between human phenotypes and genetic variations. Thousands of GWAS summary statistics are available, covering a wide range of human traits and diseases. T2DM and prediabetes are complex, multifactorial conditions influenced by both lifestyle risk factors and genetic susceptibility (5). While GWAS has identified various genetic variants associated with an increased risk of T2DM across different populations (6, 7), studies specifically targeting genetic variants linked to prediabetes remain limited, particularly in Chinese populations. This study aims to contribute to addressing this gap by exploring genetic variants associated with prediabetes risk. From a pathophysiological perspective, major procedures in the progression to T2DM include dysfunction of β-cells and insulin resistance (8). Similarly, prediabetes is characterized by increased inflammation and islet dysfunction (9), emphasizing the importance of assessing islet function indicators in individuals with prediabetes. Consequently, we also explored the associations between genetic variations and clinical indicators of islet function in this study.

Melatonin receptor 1B (*MTNR1B*), a member of the G protein-coupled receptor family encoded by the *MTNR1B* gene, was initially identified as the regulator of glucose levels and insulin secretion binding with melatonin (10). Over the past decade, GWAS studies have identified several risk variants in *MTNR1B* associated with impaired glucose regulation and related conditions. The leading variant, rs10830963 (11), has been linked to T2DM (12), diabetic peripheral neuropathy (13), gestational diabetes mellitus (14), HbA1c (15), and insulin secretion (16). Similarly, rs2166706 has been associated with elevated plasma glucose levels, reduced islet function, and an increased risk of T2DM (17), while rs1447352 has been linked to fasting plasma glucose levels (18). Another variant, rs724030, which is in moderate linkage disequilibrium with rs10830963, rs2166706, and rs1447352 (r² = 0.61, 0.23, and 0.23, respectively), has been associated with T2DM (12, 19), HbA1c (15, 20), and islet function (16) in European populations. However, the role of rs724030 in individuals with prediabetes, a condition with a high risk of progressing to T2DM, remains unexplored, particularly in East Asian populations. To address this gap, we selected rs724030 as a representative locus to investigate its genetic association with prediabetes risk and related clinical indicators.

Therefore, our study aimed to investigate the relationships between rs724030 in *MTNR1B* and clinical features in both NGT and IFG/IGT individuals among Chinese people, including plasma glucose and serum insulin levels during OGTT, islet function, and islet resistance. Furthermore, after stratifying by WHR, we explore sex dimorphisms in the genetic effects of rs724030 in *MTNR1B* on islet function.

## Materials and methods

### Study design and participants

This study includes unrelated 3415 glucose-tolerant healthy and 1744 IFG/IGT individuals, and their clinical characteristics are shown in **Supplementary Table S1**. This study included participants from Nanjing, one of the 25 communities in the REACTION study (21). Participants with a history of other diseases, positive thyroid peroxidase, or thyrotropin receptor antibodies were excluded. All participants measured plasma glucose and serum insulin levels at fasting, 30, and 120 minutes after a standard 75-g OGTT. Based on the WHO criteria (2), IGT (2-h glucose 7.8–11.0 mol/L) and IFG (fasting glucose 6.1–6.9 mmol/L) are commonly used to define prediabetes. NGT is defined as fasting glucose <6.1 mmol/L and 2-h glucose < 7.8 mol/L. The study population was determined to be Chinese Han by questionnaire. All samples were collected with appropriate informed consent from all participants and/or their guardians in a written way. Approval for the study was granted from the relevant ethics committees in the First Affiliated Hospital of Nanjing Medical University. All participants provided written informed consent at the time of recruitment.

### Study measurements

The homeostasis model assessment of β-cell function (HOMA-β), homeostasis model assessment of insulin resistance (HOMA-IR), IGI, CIR, DIo, and Matsuda’s insulin sensitivity index (ISI_Matsuda_) were calculated according to the formula, and methods in **Supplementary Table S4**. Plasma glucose was measured with the hexokinase method (AU5400, Olympus). An insulin radioimmunoassay kit measured serum insulin levels (BNIBT, China). The chemiluminescence method was used to evaluate serum C-peptide levels and serum lipid levels such as high-density lipoprotein cholesterol (HDL), low-density lipoprotein cholesterol (LDL), total cholesterol (TC), and triglycerides (TG) (RocheDiagnostics, Switzerland). In addition, a questionnaire was used to obtain their basic information, including age, sex, height, weight, waist circumference, and hip circumference.

### Genotyping assay

Human genomic DNA was extracted using a standard method. The SNP genotyping work was performed using a custom-by-design 48-Plex SNPscanTM Kit (Cat#:G0104; Genesky Biotechnologies Inc., Shanghai, China). This kit was developed according to patented SNP genotyping technology by Genesky Biotechnologies Inc., which was based on double ligation and multiplex fluorescence polymerase chain reactions (PCR) (22). Genotyping was conducted without any knowledge regarding the subject’s case or control status. Concordance for duplicate samples, which accounted for 4% of the total samples, was >99% for all assays. The genotype distribution in healthy and pre-diabetic individuals was consistent with Hardy-Weinberg equilibrium (P > 0.05).

### Statistical analysis

Mean ± SD was used for normally distributed data and median (interquartile range) for non-normal data. Values of serum insulin, HOMA-β, HOMA-IR, IGI, CIR, DIo, and ISI_Matsuda_ were log-transformed. Under an appropriately adjusted additive model, a binary logistic regression analysis was performed for the relationship between rs724030 in *MTNR1B* and the IFG/IGT individuals. The associations between the *MTNR1B* rs724030 variant and glycemic-related quantitative traits were analyzed by multiple linear regression. All the analyses were adjusted by age, sex, and BMI. All P-values were two-sided, and P < 0.05 was considered significant. The heterogeneity was considered significant with I^2^ >75% and P<0.05. Statistical analyses were performed using STATA 18.0.

## Results

### *MTNR1B* Rs724030 A>G variant is not associated with the risk of IFG/IGT individuals

**Supplementary Table S2** shows no association between the *MTNR1B* rs724030 variant and IFG/IGT risk in total individuals (P > 0.05). Obesity is a significant risk factor for the dysregulation of glucose metabolism. Therefore, we stratified participants by BMI according to Chinese criteria (23). However, we did not find any association between the *MTNR1B* rs724030 variant and IFG/IGT risk in any of the BMI subgroups (P > 0.05).

### *MTNR1B* Rs724030 A>G variant significantly correlates with islet function but not insulin resistance in the NGT individuals with normal BMI Levels

After stratifying people by BMI, we analyzed OGTT-derived indicators of glycemic-related traits in the NGT individuals and IFG/IGT individuals. As shown in **Table 1**, the *MTNR1B* rs724030 variant is associated with higher fasting and 30-minute plasma glucose levels in the NGT individuals with normal BMI levels (BMI < 24kg/m^2^) (P = 0.009 and 0.001, respectively). We further investigate the associations between this variant and islet function indices. In the NGT individuals with normal BMI levels, the G allele of this variant is significantly associated with decreased IGI and DIo (P = 0.001 and 0.007, respectively) but not associated with insulin resistance indices. Except for the similar correlation between the variant and IGI in individuals who are overweight (24 ≤ BMI < 28kg/m^2^) in the NGT individuals (P = 0.035), no other significant relationships exist in both the NGT and IFG/IGT individuals. Among individuals who are overweight in the NGT individuals, although the variant was positively correlated with glycated hemoglobin (HbA1c) levels (P = 0.003), this correlation was lower with significant heterogeneity to the individuals with normal BMI levels (P_het_ = 0.037). Similarly, among individuals who are obese (BMI ≥ 28kg/m^2^) in the NGT individuals, this variant had a negative association with fasting insulin levels and HOMA-β (P = 0.016 and 0.003), but these associations were also lower due to significant heterogeneity to the individuals with normal BMI levels (P_het_ = 0.039 for fasting insulin levels, P_het_ = 0.022 for HOMA-β).

**Table 1:** The associations between the *MTNR1B* rs724030 variant and glycemic quantitative traits, islet function/insulin resistance, and serum lipid levels stratified by BMI.

Groups	NGT			β	*P_adj_*	*P_het_*	*I^2^* (%)	IFG/IGT			β	*P_adj_*	*P_het_*	*I^2^* (%)
Traits	AA	AG	GG					AA	AG	GG				
BMI<24kg/m2														
n	648	946	385					223	364	130				
Plasma glucose (mmol/l)														
Fasting	5.27 ± 0.36	5.31 ± 0.35	5.32 ± 0.35	0.029	0.009**			5.74 ± 0.52	5.72 ± 0.50	5.77 ± 0.51	0.010	0.715		
30 min post OGTT	8.36 ± 1.42	8.51 ± 1.42	8.65 ± 1.41	0.153	0.001**			10.07 ± 1.43	10.12 ± 1.43	10.27 ± 1.25	0.086	0.251		
120 min post OGTT	6.08 ± 0.97	6.05 ± 0.97	6.06 ± 0.97	-0.012	0.681			8.51 ± 1.24	8.48 ± 1.28	8.63 ± 1.23	0.034	0.614		
HbA1c(%)	5.61 ± 0.37	5.62 ± 0.38	5.62 ± 0.36	0.005	0.675			5.80 ± 0.35	5.79 ± 0.49	5.82 ± 0.41	0.001	0.964		
Serum insulin (pmol/l)														
Fasting	9.44(7.20,12.66)	9.27(6.81,12.21)	9.22(7.11,11.87)	-0.010	0.499			9.66(7.01,13.20)	6.86(9.40,12.24)	9.60(6.27,12.22)	-0.031	0.222		
30 min post OGTT	53.20(35.34,83.00)	50.93(34.19,78.80)	51.85(35.54,73.56)	-0.032	0.093			46.94(32.32,73.29)	44.92(30.33,70.01)	44.53(29.61,61.33)	-0.055	0.100		
120 min post OGTT	38.49(25.39,57.11)	37.11(23.58,58.22)	37.45(23.89,53.51)	-0.018	0.374			70.59(45.30,101.20)	60.99(42.71,88.70)	72.94(41.88,100.43)	-0.007	0.827		
Islet function														
HOMA-β	106.88(79.75,150.73)	101.68(76.22,145.35)	100.82(77.69,140.63)	-0.028	0.082			89.17(61.79,118.15)	87.70(61.06,120.30)	80.12(57.54,118.22)	-0.036	0.191		
IGI	8.74(15.12,26.52)	13.89(7.99,24.12)	13.62(7.61,21.35)	-0.090	0.001**			8.98(5.08,14.72)	8.02(4.95,14.17)	7.26(4.57,12.25)	-0.085	0.053		
DIo	1.63(0.91,2.93)	1.54(0.82,2.66)	1.47(0.82,2.39)	-0.080	0.007**			0.93(0.48,1.64)	0.89(0.52,1.60)	0.83(0.47,1.51)	-0.053	0.256		
Insulin resistance														
HOMA-IR	2.23(1.66,3.00)	2.19(1.60,2.92)	2.18(1.65,2.88)	-0.005	0.757			2.40(1.80,3.43)	2.37(1.77,3.10)	2.40(1.60,3.28)	-0.030	0.266		
ISI_Matsuda_	0.14(3.99,5.89)	3.89(0.14,6.16)	3.76(0.11,5.68)	-0.104	0.105			3.90(2.94,5.49)	4.28(3.16,5.75)	4.19(2.97,5.60)	0.025	0.269		
serum lipid levels														
HDL	1.42(1.18,1.68)	1.40(1.19,1.62)	1.41(1.20,1.67)	0.005	0.652			1.36(1.15,1.58)	1.36(1.16,1.58)	1.37(1.10,1.61)	-0.009	0.615		
LDL	2.69(2.25,3.20)	2.69(2.24,3.20)	2.68(2.23,3.23)	0.008	0.741			2.82(2.20,3.30)	2.77(2.32,3.28)	2.94(2.38,3.49)	0.084	0.050		
TC	4.78(4.22,5.40)	4.78(4.10,5.45)	4.76(4.16,5.52)	0.001	0.980			4.88(4.22,5.59)	4.87(4.28,5.57)	5.09(4.36,5.66)	0.051	0.349		
TG	1.07(0.79,1.45)	1.04(0.78,1.41)	0.98(0.78,1.34)	-0.022	0.123			1.21(0.89,1.75)	1.22(0.91,1.70)	1.37(1.03,1.87)	0.027	0.317		
24≤BMI<28kg/m2														
n	407	585	193					277	361	153				
Plasma glucose (mmol/l)														
Fasting	5.37 ± 035	5.37 ± 0.35	5.43 ± 0.31	0.025	0.075	0.839	0.0	5.77 ± 0.50	5.81 ± 0.52	5.86 ± 0.50	0.042	0.089	0.380	0.0
30 min post OGTT	8.68 ± 1.34	8.71 ± 1.44	8.93 ± 1.36	0.108	0.060	0.537	0.0	10.13 ± 1.28	10.12 ± 1.34	10.15 ± 1.47	0.007	0.918	0.428	0.0
120 min post OGTT	6.30 ± 0.96	6.27 ± 0.95	6.23 ± 0.99	-0.023	0.563	0.830	0.0	8.72 ± 1.21	8.58 ± 1.33	8.68 ± 1.20	-0.038	0.541	0.433	0.0
HbA1c(%)	5.65 ± 0.34	5.67 ± 0.39	5.73 ± 0.36	0.044	0.003**	0.037*	77.0	5.93 ± 0.37	5.89 ± 0.43	5.87 ± 0.41	-0.027	0.173	0.325	0.0
Serum insulin (pmol/l)														
Fasting	11.04(8.13,14.82)	10.26(8.01,13.46)	10.68(8.11,15.59)	-0.019	0.342	0.733	0.0	11.04(8.28,14.93)	10.72(7.73,14.02)	11.17(8.44,14.02)	-0.016	0.521	0.664	0.0
30 min post OGTT	68.17(46.29,108.60)	62.75(41.23,95.78)	64.71(42.69,96.27)	-0.041	0.106	0.783	0.0	63.30(40.07,93.55)	57.67(39.09,89.45)	55.78(35.28,85.18)	-0.056	0.068	0.976	0.0
120 min post OGTT	48.34(30.63,60.17)	46.10(29.15,71.74)	54.27(30.47,86.07)	0.033	0.231	0.136	55.0	79.08(49.93,115.75)	80.22(50.82,112.75)	86.26(58.22,115.65)	0.022	0.428	0.496	0.0
Islet function														
HOMA-β	119.33(88.37,166.07)	111.95(84.84,150.81)	115.47(81.36,159.49)	-0.034	0.108	0.834	0.0	99.24(71.63,136.35)	96.83(65.03,130.45)	100.00(68.27,131.01)	-0.035	0.178	0.981	0.0
IGI	18.52(11.05,31.66)	16.98(9.88,29.01)	16.89(8.63,26.42)	-0.074	0.035*	0.705	0.0	11.92(7.26,18.83)	11.02(6.59,18.30)	10.98(5.82,17.62)	-0.053	0.182	0.588	0.0
DIo	1.69(0.91,3.12)	1.68(0.93,2.79)	1.51(0.79,2.52)	-0.055	0.144	0.593	0.0	1.07(0.65,1.88)	1.08(0.59,1.69)	1.04(0.52,0.48)	-0.037	0.387	0.796	0.0
Insulin resistance														
HOMA-IR	2.64(1.95,3.57)	2.46(1.89,3.26)	2.62(1.89,3.75)	-0.014	0.486	0.716	0.0	2.81(2.05,3.88)	2.75(1.95,3.67)	2.88(2.13,3.66)	-0.009	0.737	0.569	0.0
ISI_Matsuda_	2.76(0.07,4.23)	2.53(0.07,4.92)	2.63(0.07,4.37)	-0.075	0.419	0.803	0.0	3.40(2.55,4.39)	3.49(2.64,4.65)	3.31(2.57,4.19)	0.007	0.754	0.557	0.0
serum lipid levels														
HDL	1.22(1.02,1.42)	1.24(1.05,1.46)	1.26(1.05,1.41)	0.012	0.316	0.636	0.0	1.20(1.04,1.41)	1.21(1.01,1.44)	1.22(1.04,1.42)	-0.008	0.581	0.956	0.0
LDL	2.78(2.32,3.25)	2.73(2.22,3.25)	2.76(2.20,3.23)	-0.032	0.318	0.318	0.0	2.81(2.34,3.35)	2.83(2.30,3.34)	2.84(2.32,3.41)	-0.001	0.989	0.151	51.6
TC	4.77(4.19,5.32)	4.65(4.00,5.32)	4.77(3.99,5.42)	-0.043	0.295	0.397	0.0	4.81(4.34,5.57)	4.85(4.22,5.58)	4.78(4.20,5.57)	-0.011	0.833	0.408	0.0
TG	1.32(1.00,1.83)	1.25(0.90,1.75)	1.33(0.94,1.86)	-0.018	0.376	0.935	0.0	1.51(1.16,2.26)	1.45(1.12,2.13)	1.55(1.19,2.00)	-0.006	0.788	0.356	0.0
BMI≥28kg/m2														
n	84	134	33					74	121	41				
Plasma glucose (mmol/l)														
Fasting	5.34 ± 0.38	5.44 ± 0.34	5.45 ± 0.33	0.056	0.104	0.454	0.0	5.90 ± 0.50	5.93 ± 0.47	5.88 ± 0.54	-0.001	0.979	0.871	0.0
30 min post OGTT	8.73 ± 1.33	9.06 ± 1.29	9.25 ± 1.45	0.209	0.099	0.676	0.0	10.29 ± 1.39	10.33 ± 1.51	10.90 ± 1.49	-0.062	0.660	0.354	0.0
120 min post OGTT	6.39 ± 1.03	6.39 ± 0.93	6.43 ± 0.93	0.007	0.945	0.850	0.0	8.45 ± 1.48	8.72 ± 1.22	8.74 ± 1.25	0.115	0.346	0.563	0.0
HbA1c(%)	5.64 ± 0.44	5.71 ± 0.40	5.77 ± 0.38	0.044	0.259	0.335	0.0	5.94 ± 0.37	5.92 ± 0.40	6.03 ± 0.38	0.030	0.419	0.494	0.0
Serum insulin (pmol/l)														
Fasting	13.66(9.98,18.77)	13.98(9.68,18.37)	11.65(8.32,15.84)	-0.112	0.016*	0.039*	76.6	11.83(8.56,15.50)	12.14(9.34,17.17.47)	13.25(10.44,16.06)	0.051	0.252	0.110	60.9
30 min post OGTT	87.09(64.96,128.10)	87.72(57.62,124.90)	77.39(47.73,97.33)	-0.079	0.140	0.409	0.0	77.12(43.06,106.13)	66.78(47.11,100.25)	72.74(44.55,92.12)	-0.054	0.329	0.993	0.0
120 min post OGTT	60.06(36.92,97.93)	56.44(39.52,87.39)	48.78(38.54,76.10)	-0.083	0.169	0.309	3.3	79.52(52.95,12.15)	86.90(59.51,123.55)	94.34(66.55,129.90)	0.096	0.097	0.120	58.6
Islet function														
HOMA-β	153.30(113.72,204.54)	148.42(97.72,195.77)	124.73(80.02,161.98)	-0.145	0.003**	0.022*	80.8	95.96(74.92,136.07)	104.53(78.60,142.60)	108.63(77.00,158.21)	0.054	0.239	0.094	64.3
IGI	22.92(14.52,32.81)	20.99(13.29,28.89)	17.37(11.71,28.15)	-0.120	0.072	0.685	0.0	14.62(8.10,22.10)	12.56(7.80,21.52)	13.26(6.99,20.61)	-0.075	0.286	0.913	0.0
DIo	1.63(1.03,2.73)	1.56(1.07,2.41)	1.69(1.33,2.04)	-0.008	0.910	0.346	0.0	1.23(0.74,1.95)	1.16(0.65,1.68)	1.07(0.53,1.57)	-0.127	0.095	0.410	0.0
Insulin resistance														
HOMA-IR	3.29(2.28,4.48)	3.38(2.38,4.50)	2.75(2.04,3.78)	-0.101	0.034*	0.055	72.8	3.12(2.31,4.13)	3.17(2.32,4.68)	3.64(2.72,4.25)	0.051	0.272	0.133	55.7
ISI_Matsuda_	2.21(0.03,3.35)	2.30(0.05,3.34)	2.97(0.06,4.53)	0.287	0.188	0.086	66.0	3.11(2.44,3.92)	3.04(2.21,3.92)	2.86(2.40,3.68)	-0.033	0.388	0.195	40.6
serum lipid levels														
HDL	1.15(1.00,1.44)	1.20(1.01,1.35)	1.07(0.97,1.20)	-0.032	0.238	0.207	37.1	1.15(1.01,1.37)	1.20(1.05,1.40)	1.23(1.03,1.44)	0.039	0.092	0.102	62.6
LDL	2.73(2.24,3.24)	2.73(2.27,3.32)	2.48(2.21,2.87)	-0.034	0.632	0.577	0.0	2.78(2.24,3.54)	2.82(2.41,3.32)	2.79(2.40,3.43)	-0.021	0.768	0.207	37.2
TC	4.69(3.99,5.18)	4.72(4.16,5.28)	4.40(3.85,5.01)	-0.031	0.726	0.735	0.0	4.82(4.09,5.61)	4.83(4.30,5.47)	4.74(4.13,5.40)	-0.005	0.956	0.589	0.0
TG	1.55(1.03,2.08)	1.47(1.06,2.14)	1.65(1.04,2.04)	0.027	0.597	0.356	0.0	1.63(1.24,2.29)	1.69(1.23,2.31)	1.62(1.31,2.02)	0.002	0.965	0.627	0.0

For glycemic quantitative traits, linear regression analysis was used, adjusted with age, sex, BMI. Values of serum insulin, HOMA-β, HOMA-IR, IGI, DIo, and ISI_Matsuda_ were logarithmically transformed. For serum lipid levels, linear regression analysis was used, adjusted with age, sex, BMI, and TG was logarithmically transformed. NGT, normal glucose tolerance; IFG, impaired fasting glucose; IGT, impaired glucose tolerance; BMI, body mass index; HbA1c, Hemoglobin A1c; OGTT, oral glucose tolerance test; HOMA-β, homeostasis model assessment of β-cell function; IGI, insulinogenic index; DIo, oral disposition index; HOMA-IR, homeostasis model assessment of insulin resistance; ISI_Matsuda_, Matsuda’s insulin sensitivity index; HDL, high-density lipoprotein; LDL, low-density lipoprotein; TC, total cholesterol; TG, triglyceride. All P values were two-tailed and P < 0.05 was been considered as significant. *P < 0.05; **P < 0.01. P_het_, P values for heterogeneity. The heterogeneity was considered significant with I^2^ > 75% and P < 0.05.

### Significant sexual heterogeneities for the effect size on the associations between *MTNR1B* Rs724030 variant and islet function after stratifying by the WHR in the NGT individuals

We stratify all participants by WHR according to central obesity diagnosis criteria in WHO consultation (24). We grouped WHR according to 0.85 and assumed that individuals with WHR greater than 0.85 had a higher risk of abdominal obesity. As shown in **Table 2**, in the NGT individuals, significant associations were found between this variant in *MTNR1B* rs724030 and IGI, CIR, and DIo (All P < 0.001) in female individuals whose WHR are more remarkable than 0.85, and these associations had significant heterogeneity (P_het_ = 0.009, 0.030, and 0.049, respectively) to the corresponding male subgroup. This suggests that the effect of this variant on islet function exhibits sex dimorphism, with differing effect sizes between males and females. In the NGT individuals, this variant did not correlate with islet function/resistance indices among individuals whose WHR is less than or equal to 0.85. We also found no associations between this variant and related indices in the IFG/IGT individuals as shown in **Table 3**.

**Table 2:** The associations between the *MTNR1B* rs724030 variant and islet function/insulin resistance in the NGT individuals stratified by WHR.

Groups	Male			β	*P_adj_*	Female			β	*P_adj_*	*P_het_*	*I^2^* (%)
Traits	AA	AG	GG			AA	AG	GG				
WHR ≤ 0.85												
HOMA-β	104.14(70.08,139.70)	97.10(73.10,125.66)	88.73(68.37,117.52)	-0.049	0.360	116.36(84.34, 153.17)	105.58(80.74,150.23)	103.62(78.50,147.02)	-0.037	0.068	0.836	0.0
IGI	12.74(6.20,22.09)	10.26(6.33,16.78)	9.42(4.59,17.38)	-0.074	0.430	16.42(9.55,29.27)	16.83(9.45,27.15)	14.81(8.45,26.96)	-0.046	0.178	0.778	0.0
CIR	125.77(76.31,209.87)	101.07(69.22,166.89)	102.08(54.20,159.88)	-0.088	0.242	165.35(103.98,280.42)	164.50(97.93,255.50)	141.75(89.01,260.15)	-0.057	0.046*	0.700	0.0
DIo	1.34(0.62,2.87)	1.20(0.69,2.10)	1.24(0.71,2.03)	-0.013	0.898	1.73(0.96,3.21)	1.77(1.00,3.01)	1.60(0.88,2.80)	-0.028	0.436	0.884	0.0
HOMA-IR	2.33(1.50,3.11)	2.14(1.57,2.68)	1.92(1.38,2.59)	-0.065	0.201	2.29(1.76,3.03)	2.19(1.63,3.01)	2.29(1.71,2.97)	-0.012	0.551	0.329	0.0
ISIMatsuda	4.13(0.24,6.55)	4.32(0.20,6.88)	5.03(0.35,8.11)	-0.048	0.803	3.40(0.11, 5.44)	3.63(0.10,5.77)	3.61(0.010,5.52)	-0.015	0.860	0.873	0.0
WHR>0.85												
HOMA-β	109.41(80.58,163.71)	108.04(79.35,155.64)	102.51(75.19,156.65)	-0.028	0.250	119.20(88.84,169.83)	111.35(81.50,152.72)	107.39(81.22,153.17)	-0.044	0.043*	0.631	0.0
IGI	15.34(8.96,26.21)	15.13(8.51,26.17)	14.61(8.13.23.02)	-0.040	0.319	19.85(11.55,30.97)	15.52(9.49,26.81)	15.22(8.71,22.97)	-0.181	<0.001***	0.009**	85.5
CIR	152.07(89.36,238.96)	143.21(85.79,229.91)	134.60(77.98,202.05)	-0.056	0.102	198.12(114.02,295.57)	155.15(100.54,242.72)	143.30(90.25,209.73)	-0.156	<0.001***	0.030*	78.7
DIo	1.42(0.79,2.71)	1.46(0.79,2.56)	1.51(0.87,2.28)	-0.038	0.383	1.75(1.02,3.16)	1.64(0.86,2.68)	1.47(0.82,2.30)	-0.151	<0.001***	0.049*	74.1
HOMA-IR	2.45(1.86,3.46)	2.54(1.84,3.44)	2.55(1.88,3.60)	0.007	0.785	2.47(1.81,3.51)	2.41(1.83,3.33)	2.30(1.73,3.32)	-0.025	0.242	0.328	0.0
ISIMatsuda	2.99(0.08,5.19)	3.16(0.09,5.25)	2.57(0.07,4.94)	-0.047	0.651	3.38(0.08,5.12)	2.86(0.07,4.88)	3.05(0.09,5.15)	-0.149	0.116	0.466	0.0

Data are expressed as median (interquartile range). NGT, normal glucose tolerance; WHR, waist-to-hip ratio; HOMA-β, homeostasis model assessment of β-cell function; IGI, insulinogenic index; CIR, corrected insulin response; DIo, oral disposition index; HOMA-IR, homeostasis model assessment of insulin resistance; ISI_Matsuda_, Matsuda’s insulin sensitivity index; All P values were two-tailed and P < 0.05 was been considered as significant. *P < 0.05; **P < 0.01; ***P < 0.001. P_het_, P values for heterogeneity. The heterogeneity was considered significant with I^2^ > 75% and P < 0.05.

**Table 3:** The associations between the *MTNR1B* rs724030 variant and islet function/insulin resistance in the IFG/IGT individuals stratified by WHR.

Groups	Male			β	*P_adj_*	Female			β	*P_adj_*	*P_het_*	*I^2^* (%)
Traits	AA	AG	GG			AA	AG	GG				
WHR ≤ 0.85												
HOMA-β	79.93(51.07,98.83)	70.53(52.41,96.77)	61.76(36.54,109.91)	-0.025	0.814	95.86(64.61,132.14)	95.06(68.65.135.05)	89.00(68.85,120.93)	-0.017	0.614	0.941	0.0
IGI	6.81(3.83,11.88)	5.17(3.56,10.11)	5.07(3.97,11.21)	0.025	0.863	9.40(5.04,15.98)	9.40(5.49,17.13)	8.33(4.93,13.76)	-0.055	0.319	0.616	0.0
CIR	61.93(31.85,94.25)	55.30(38.38,88.24)	52.13(30.24,99.55)	0.025	0.838	87.03(52.89,141.26)	89.45(56.45,138.94)	76.02(44.71,118.18)	-0.072	0.124	0.463	0.0
DIo	0.74(0.40,1.43)	0.64(0.38,1.46)	0.72(0.62,1.56)	0.085	0.625	0.93(0.49,1.97)	0.96(0.55,1.65)	0.83(0.47,1.46)	-0.038	0.505	0.508	0.0
HOMA-IR	2.40(1.63,3.05)	2.05(1.58,2.56)	1.88(1.06,3.20)	-0.072	0.483	2.57(1.80,3.53)	2.62(1.81,3.39)	2.61(1.91,3.49)	-0.017	0.607	0.614	0.0
ISIMatsuda	4.81(3.32,6.12)	5.11(3.93,6.51)	5.29(3.33,7.18)	0.036	0.617	3.90(2.99,5.31)	3.77(2.95,5.27)	3.70(2.69,4.87)	-0.006	0.848	0.597	0.0
WHR>0.85												
HOMA-β	91.92(67.34,126.87)	89.45(61.22,123.02)	92.45(59.96,125.62)	-0.033	0.291	101.75(72.88,134.50)	96.58(68.45,131.23)	93.83(70.19,135.70)	-0.013	0.624	0.626	0.0
IGI	10.69(6.37,18.12)	10.21(5.65,16.57)	9.65(5.02,17.77)	-0.099	0.055	12.69(7.25,19.57)	10.79(6.86,17.67)	10.96(6.06,17.63)	-0.072	0.070	0.685	0.0
CIR	92.48(57.12,145.28)	84.71(48.99,138.04)	78.34(49.96,144.28)	-0.062	0.148	108.04(66.82,167.30)	94.68(62.04,152.12)	94.02(53.64,145.13)	-0.068	0.051	0.918	0.0
DIo	1.02(0.53,1.69)	0.90(0.55,1.63)	1.01(0.47,1.69)	-0.076	0.169	1.13(0.69,1.78)	1.09(0.68,1.74)	1.04(0.53,1.71)	-0.079	0.068	0.962	0.0
HOMA-IR	2.82(2.14,3.98)	2.59(1.93,3.82)	2.88(2.00,3.88)	-0.018	0.574	2.78(1.91,3.79)	2.74(2.00,3.62)	2.75(2.00,3.64)	0.015	0.576	0.430	0.0
ISIMatsuda	3.38(2.61,4.43)	3.77(2.70,5.03)	3.37(2.63,4.90)	0.031	0.249	3.28(2.66,4.62)	3.43(2.66,4.62)	3.39(2.71,4.40)	-0.002	0.916	0.337	0.0

Data are expressed as median (interquartile range). IFG, impaired fasting glucose; IGT, impaired glucose tolerance; WHR, waist-to-hip ratio; HOMA-β, homeostasis model assessment of β-cell function; IGI, insulinogenic index; CIR, corrected insulin response; DIo, oral disposition index; HOMA-IR, homeostasis model assessment of insulin resistance; ISI_Matsuda_, Matsuda’s insulin sensitivity index; All P values were two-tailed and P < 0.05 was been considered as significant.. P_het_, P values for heterogeneity. The heterogeneity was considered significant with I^2^ > 75% and P < 0.05.

### *MTNR1B* Rs724030 A>G variant is not associated with insulin clearance in the NGT individuals

We calculated the insulin clearance rate according to the method of a previously published article (25). Endogenous insulin clearance during the OGTT was assessed by CpAUC120/InsAUC120 (25). We found that the *MTNR1B* rs724030 variant was not significantly associated with insulin clearance(All P > 0.05), as shown in **Supplementary Table S3**.

## Discussion

During the last ten years, several studies have demonstrated that different variants in *MTNR1B* are associated with an increased risk of T2DM. For instance, Chambers, J.C. et al. found that the G allele of rs2166706 in *MTNR1B* is associated with an increased risk of T2DM in individuals of Asian Indian ancestry (17). Later, B. F. et al. found that the T allele of rs1387153 in *MTNR1B* can increase the risk of T2DM in individuals of European ancestry (26). Over the next few years, the researchers successively identified the association between the G allele of rs10830963 in *MTNR1B* and the increased risk of T2DM among people of different ethnicities (27, 28). Our study found no association between the *MTNR1B* rs724030 variant and the risk of predibetes, at least in the Chinese population. The increased risk of prediabetes likely arises from the combined influence of genetic and environmental factors. A single genetic variation may be difficult to fully explain the risk, highlighting the need to consider gene-environment interactions.

*MTNR1B* is a glycemic-related gene. Genetic variants in *MTNR1B* associated with fasting plasma glucose levels are increasingly known across ethnic groups (29). Previous studies have established that some SNPs in *MTNR1B* are associated with higher fasting plasma glucose levels, such as the rs1387153 T allele (30), the rs10830963 G allele (31), the rs1447352 G allele (18), rs2166706 G allele (17). In our study, after stratifying people by BMI status, we also found that the minor (G) allele of the variant in *MTNR1B* rs724030 was associated with a per-allele increase of 0.029 (95%CI 0.007–0.050) mmol/L and 0.153 (95%CI 0.067–0.240) mmol/L in fasting plasma glucose levels (P = 0.009) and 30-minute plasma glucose levels (P = 0.001), respectively, in the NGT individuals with normal BMI levels. It demonstrated that studies of continuous glycemic traits in the NGT individuals can improve the understanding of the mechanisms involved in β-cell function and glucose homeostasis. However, we found no association between this variant and 120-minute plasma glucose levels in all participants. Among individuals who are overweight and obese in the NGT individuals, we found no association between this variant and glycemic-related traits. At the same time, no associations were found in the IFG/IGT individuals. Therefore, we suggest that the G allele of rs724030 in *MTNR1B* may have a possible association with higher plasma glucose levels in populations with normal glucose tolerance and with normal BMI levels but not in populations in the prediabetic state. It may be a potential risk factor for abnormal glucose metabolism in healthy individuals in the future but needed to be further verified. HbA1c reflects the average plasma glucose levels over the past three months and indicates long-term glycemic status, unlike fasting plasma glucose (32). As previously shown, rs1387153 (33) and rs10830963 (34) in *MTNR1B* are associated with increased HbA1c levels. However, we found no association between rs724030 and HbA1c levels in all subgroups, the individals we studied were elderly subjects based on OGTT or ethnic heterogeneity may contribute to the inconsistent results.

Melatonin, a neurohormone, regulates the circadian rhythm by transmitting photoperiodic information from the eyes to the brain. Melatonin signaling is mainly mediated by two high-affinity receptors, MT1 and MT2, encoded by the *MTNR1A* and *MTNR1B* genes, respectively (35). MT2-mediated melatonin signaling could indirectly regulate glucose levels and insulin secretion through the brain control center of the circadian clock (30). In rodent and human islets, *MTNR1B* is expressed and co-localizes with insulin. Researchers have demonstrated that treating the pancreatic beta-cells with melatonin can worsen insulin secretion and that exogenously administered melatonin inhibits insulin secretion in rodents (36). *MTNR1B* is identified to inhibit glucose-stimulated insulin secretion through binding with melatonin and decreasing cAMP levels (10). Consistent with previous studies, herein, in the NGT individuals, we observed that subjects carrying the minor (G) allele of the variant in *MTNR1B* rs724030 tend to have lower IGI and DIo levels. These two indicators can measure β-cell function adjusted for insulin sensitivity and predict the development of diabetes over ten years (37). Previous studies have also demonstrated that the rs10830963 G allele in *MTNR1B* is associated with decreased insulin secretion measured as CIR, AUC_Ins_/AUC_Gluc_ (38), peak insulin response, acute insulin response (AIR), disposition index (DI) (16). Similarly, Palmer, N.D. et al. also found that rs1387153 T allele in *MTNR1B* affects insulin secretion pathways reflected by AIR and disposition index (DI) (39). It demonstrated that variants in *MTNR1B* can influence β-cell function. In our study, we found that the G allele of rs724030 in *MTNR1B* was associated with decreased IGI (P = 0.001) and DIo (P = 0.007) in the NGT individuals, especially in those NGT participants with normal BMI levels. It indicates that this indicator can reflect the deterioration of β-cell function and predict the possibility of impaired plasma glucose regulation. However, similar association wasn’t found in obese subgroups in the NGT individuals, and no associations in the IFG/IGT individuals.

It is well known that obesity has continued to increase for three decades worldwide (40) and has become a tremendous public health concern globally. Obesity can be categorized as general and abdominal obesity, while abdominal obesity has been established to be associated with T2DM (41). Among the conventional indicators of obesity, waist circumference and WHR are used to reflect abdominal obesity, and BMI is used to reflect general obesity. Previous studies have identified that increased waist circumference and WHR is associated with higher T2DM risk (42). Probably because the distribution of abdominal fat correlates with increased insulin resistance. Consistent with previous observations, in the NGT individuals, we found that the minor (G) allele of the variant in *MTNR1B* rs724030 is associated with decreased IGI, CIR, and DIo, especially in female subjects with WHR greater than 0.85. Moreover, this correlation was specific due to the significant heterogeneity in the corresponding male subgroup. Possible explanations for this finding include the following: Differences in sex hormone levels, such as estrogen and testosterone, may influence fat distribution, with women tending to accumulate fat in the abdomen, hips, and thighs. Additionally, men and women differ in insulin secretion, sensitivity, and glucose metabolism, which could result in sex-specific effects of the same SNP. Lastly, variations in diet, exercise, and lifestyle habits between men and women may amplify the SNP’s sex-specific effects through gene-environment interactions. At the same time, we did not find any association between this variant and islet function/islet resistance indices in the IFG/IGT individuals after stratifying by WHR. We hypothesize that abnormal glucose regulation may obscure the effects of other factors on islet function, such as genetic variants.

The strengths of this study included that OGTT was used to recognize the status of glucose regulation in every group population, which allowed us to identify the association between the variant and postprandial indices. More importantly, this is the first study to investigate the associations of this variant with clinical features and islet function/resistance in the Chinese Han population. The present study also has some inevitable limitations. For example, findings of this study are unlikely to be explained by a single genetic variant alone. A limitation of this study is the lack of consideration for potential confounding environmental factors, such as diet, physical activity, and other lifestyle behaviors, which are known to significantly influence glucose levels and insulin resistance. While we focused on genetic associations, the absence of detailed data on these variables limits our ability to fully disentangle genetic effects from environmental influences. Future studies should integrate comprehensive assessments of lifestyle factors to provide a more holistic understanding of the interplay between genetics and environment in determining metabolic outcomes. While this study primarily focuses on the analysis of *MTNR1B* gene variants and their associations with prediabetes risk, we acknowledge the limitation of not including variants from other genes that may also contribute to prediabetes risk. Future studies including a broader range of genetic variants would provide a more comprehensive understanding of the genetic architecture underlying prediabetes susceptibility.

In conclusion, we found that the *MTNR1B* rs724030 variant is correlated with the increase of fasting and 30-minute plasma glucose levels in the NGT individuals with normal BMI levels. Importantly, we also found that this variant is associated with the impairment of islet function measured as IGI and DIo in the same subgroup. Moreover, the current study provides, for the first time, insights into the sex dimorphisms of the effects of the *MTNR1B* rs724030 variant on impairing islet function after stratifying by WHR in the NGT individuals. These discoveries provide a basis for accurately assessing islet function in healthy Chinese populations and offer a new perspective on precision prevention. Future studies must further explain the BMI-specific differences and sex dimorphisms in this variant’s impact on islet function.

## Data Availability

The original contributions presented in the study are included in the article/**Supplementary Material**, further inquiries can be directed to the corresponding authors.
