# Supplementary material for: The MTNR1B Rs724030 variant is associated with islet function and women waist-to-hip ratio in healthy subjects
Source: Front Endocrinol (Lausanne). 2025 Jan 16;15:1398687. doi: 10.3389/fendo.2024.1398687 (PMC11779613; doi:10.3389/fendo.2024.1398687)
Supplement: Supplementary file 1 [file DataSheet1.pdf]

Supplementary Material

Table S1 Characteristics of healthy controls and prediabetes recruited for the study

	**NGT**	**IFG/IGT**
n	3415	1744
Sex (male/female)	1089/2326	623/1121**
Age (years)	54.95±9.08	58.27±9.24***
Weight(kg)	1.61±0.08	64.02±10.24***
Height(m)	61.59±10.05	1.61±0.08***
BMI (kg/m^2)	23.65±2.97	24.78±3.04***
Waist circumference(cm)	81.91±8.86	85.61±8.86***
Hip circumference(cm)	95.10±6.16	96.79±6.48***
WHR	0.86±0.07	0.88±0.06***
Systolic blood pressure(mmHg)	125.47±16.57	132.26±16.77***
Diastolic blood pressure(mmHg)	76.54±10.44	78.88±10.55***
Pulse(bpm)	77.00±10.61	78.77±11.35***
**Plasma glucose (mmol/l)**		
Fasting	5.33±0.35	5.79±0.51***
30 min post OGTT	8.61±1.41	10.15±1.38***
120 min post OGTT	6.16±0.97	8.59±1.27***
HbA1c (%)	5.64±0.38	5.87±0.40***
**Serum insulin (mIU/L)**		
Fasting	9.93(7.49,13.46)	10.40(7.61,13.95)*
30 min post OGTT	57.80(38.83,89.20)	54.85(35.59,85.96)***
120 min post OGTT	41.89(26.72,64.63)	74.79(48.84,108.20)***
**Islet function**		
HOMA-β	109.17(81.11,153.86)	93.60(66.28,129.44)***
IGI	15.63(9.11,26.58)	10.31(5.88,17.26)***
CIR	153.88(94.35,245.77)	89.26(55.39,144.62)***
DIo	1.59(0.88,2.77)	1.01(0.55,1.67)***
**Insulin resistance**		
HOMA-IR	2.34(1.76,3.23)	2.67(1.92,3.62)***
$ISI_{Matsuda}$	3.32(0.09,5.35)	3.59(2.72,4.90)***
**Lipid levels (mmol/L)**		
HDL	1.32(1.11,1.56)	1.26(1.06,1.48)***
LDL	2.71(2.25,3.21)	2.81(2.32,3.35)***
TC	4.75(4.13,5.38)	4.86(4.26,5.57)***
TG	1.13(0.85,1.61)	1.41(1.05,1.97)***

Notes: Data are expressed as mean ± SD or as median (interquartile range). NGT, normal glucose tolerance; IFG, impaired fasting glucose; IGT, impaired glucose tolerance; BMI, body mass index; WHR, waist-to-hip ratio; HbA1c, Hemoglobin A1c; OGTT, oral glucose tolerance test; HOMA-β, homeostasis model assessment of β-cell function; IGI, insulinogenic index; CIR, corrected insulin response; DIo, oral disposition index; HOMA-IR, homeostasis model assessment of insulin resistance; $ISI_{Matsuda}$, Matsuda's insulin sensitivity index; HDL, high-density lipoprotein; LDL, low-density lipoprotein; TC, total cholesterol; TG, triglyceride. All P values were two-tailed and $P < 0.05$ was been considered as significant. *$P < 0.05$; **$P < 0.01$; ***$P < 0.001$.

Table S2 The association between the *MTNR1B* rs724030 A>G variant and IFG/IGT risk stratified by BMI

Groups	Genotype distribution			MAF		Additive model	
	**AA**	**AG**	**GG**	**Prediabetes**	**Controls**	**OR (95%CI)**	***P***
Total IFG/IGT	574	846	324	0.428		1.023(0.942-1.111)	0.585
BMI＜24	223	364	130	0.435		1.006(0.892-1.136)	0.917
24≤BMI＜28	277	361	153	0.422		1.050(0.923-1.193)	0.459
BMI≥28	74	121	41	0.430		1.153(0.883-1.505)	0.296
Total NGT	1139	1665	611		0.423		

Notes: MAF, minor allele frequency; NGT, normal glucose tolerance; IFG, impaired fasting glucose; IGT, impaired glucose tolerance; BMI, normal:BMI< 24kg/m^2; overweight:24kg/m^2≤BMI< 28kg/m^2; obese:BMI≥ 28kg/m^2. All P values were two-tailed and P < 0.05 was been considered as significant. *P < 0.05; **P < 0.01; ***P < 0.001.

Table S3 The association between the *MTNR1B* rs724030 variant and insulin clearance

	Genotype distribution				
	**AA**	**AG**	**GG**	**β**	P_{adj}
n (men/women)	137(76/61)	179(94/85)	72(35/37)		
CpAUC (nmol/L)	291.55±76.94	296.02±74.55	284.38±76.64	-1.804	0.720
InsAUC (nmol/L)	53.13±25.55	52.48±25.58	49.75±24.67	-2.075	0.206
CpAUC120/InsAUC120	6.41±2.57	6.46±2.18	6.53±2.22	0 .034	0.115

Notes: Data are expressed as mean±SD. All P values were two-tailed and P < 0.05 was been considered as significant. *P < 0.05; **P < 0.01; ***P < 0.001.

Table S4 Calculation of glycemic indexes

Trait	Measurement or calculation
HOMA-β [1]	(20 × fasting serum insulin (μU/mL))/(fasting plasma glucose (mmol/l) - 3.5)
HOMA-IR [2]	(fasting plasma glucose (mmol/l) × fasting serum insulin (μU/mL)) / 22.5
$ISI_{Matsuda}$ [3]	10,000/√(fasting plasma glucose (mg/dl) × fasting serum insulin(μU/mL)) ×(mean plasma glucose (mg/dl) × mean serum insulin(μU/mL) during OGTT))
CIR [4]	((serum insulin 30 min (pmol/l)/6.945) × 100)/(plasma glucose 30 min(mmol/l)×(plasma glucose 30 min(mmol/l)−3.89))
IGI [5]	change in insulin during the early 30 min(ΔI_{0-30}, μU/ml) / change in glucose during the early 30 min(ΔG_{0-30}, mmol/l)
DIo [5]	IGI/ fasting serum insulin (μU/ml)

References

[1] Matthews D, Hosker J, Rudenski A, Naylor B, Treacher D, Turner R. Homeostasis Model Assessment: Insulin Resistance and Beta-Cell Function from Fasting Plasma Glucose and Insulin Concentrations in Man. *Diabetologia* (1985) 28:412-9. doi:10.1007/BF00280883

[2] Radziuk J. Insulin Sensitivity and Its Measurement: Structural Commonalities among the Methods. *J Clin Endocrinol Metab* (2000) 85:4426-33. doi:10.1210/jcem.85.12.7025

[3] Matsuda M, DeFronzo R. Insulin Sensitivity Indices Obtained from Oral Glucose Tolerance Testing: Comparison with the Euglycemic Insulin Clamp. *Diabetes Care* (1999) 22:1462-70. doi:10.2337/diacare.22.9.1462

[4] Sluiter W, Erkelens D, Reitsma W, Doorenbos H. Glucose Tolerance and Insulin Release, a Mathematical Approach I. Assay of the Beta-Cell Response after Oral Glucose Loading. *Diabetes* (1976) 25:241-4. doi:10.2337/diab.25.4.241

[5] Utzschneider K, Prigeon R, Faulenbach M, Tong J, Carr D, Boyko E, et al. Oral Disposition Index Predicts the Development of Future Diabetes above and Beyond Fasting and 2-H Glucose Levels. *Diabetes Care* (2009) 32:335-41. doi:10.2337/dc08-1478
